# Cardiometabolic risk in young adults with depression and evidence of inflammation: A birth cohort study

**DOI:** 10.1016/j.psyneuen.2020.104682

**Published:** 2020-06

**Authors:** Benjamin I. Perry, Bianca P. Oltean, Peter B. Jones, Golam M. Khandaker

**Affiliations:** a Department of Psychiatry, University of Cambridge School of Clinical Medicine, Cambridge, UK; b Cambridgeshire and Peterborough NHS Foundation Trust, Cambridge, UK

**Keywords:** Depression, Inflammation, CRP, Cardiometabolic risk factors, Cardiovascular, ALSPAC

## Abstract

**Background:**

Young adults with depression and evidence of inflammation may represent a high-risk group for cardiometabolic disorders, but studies of cardiometabolic risk in this population are scarce. We aimed to examine: (1) the prevalence of low-grade inflammation in young-adults with depression; (2) cross-sectional and longitudinal associations between cardiometabolic risk factors and depression with or without evidence of inflammation.

**Method:**

The ALSPAC birth cohort participants were assessed for depression and serum high-sensitivity C-Reactive Protein (CRP) levels at age 18, alongside cardiometabolic measures (fasting insulin, fasting plasma glucose, low-density lipoprotein, high-density lipoprotein, triglycerides, smoking, alcohol intake) at age 18 years, and body mass index at ages 9, 13 and 18 years. Low-grade inflammation was defined as CRP>3 mg/L. Multinomial regression was used to examine associations of cardiometabolic markers with depression cases with and without evidence of inflammation. Sensitivity analyses were conducted to examine for interactions between depression, inflammation and cardiometabolic traits.

**Results:**

Out of 2932 participants, 215 met ICD-10 criteria for depressive episode at age 18 years; 23 (10.7 %) had CRP>3 mg/L and 57 (26.5 %) had CRP 1−3 mg/L. Depressive episode with raised CRP (>3 mg/L) was associated with higher triglycerides (adjusted OR = 2.09; 95 % C.I., 1.35−3.24), higher BMI (adjusted OR = 1.13; 95 % C.I., 1.05−1.22) and insulin insensitivity (adjusted OR = 1.12; 95 % C.I., 1.01−1.26), and longitudinally with higher BMI at ages 9 (adjusted OR = 1.27; 95 % C.I., 1.10−1.48) and 13 (adjusted OR = 1.23; 95 % C.I., 1.09−1.38). There was evidence for interaction between BMI and CRP for the risk of depression at age 18 (adjusted OR for the interaction term = 1.56; 95 % C.I. 0.98–2.02) and between CRP and depressive symptoms for the risk of increased BMI at age 18 (adjusted β for the interaction term = 0.05; 95 % C.I. 0.00−0.12).

**Conclusions:**

A notable proportion of young adults with depression have evidence of inflammation. These individuals are at increased risk of cardiometabolic disorders. Management of cardiometabolic risk in depressed individuals with evidence of inflammation should form part of routine clinical practice.

## Highlights

• Evidence of inflammation (CRP>3 mg/L) was seen in about 10 % young-adults with depression at age 18.
• Depressed individuals with evidence of inflammation are at increased risk for cardiometabolic dysfunction.
• Raised childhood BMI is associated with depression with inflammation at age 18.
• Depression may interact with inflammation to increase risk of cardiometabolic dysfunction.

## 1 Introduction

Low-grade inflammation is implicated in pathogenesis of depression (Lee and Giuliani, 2019; Miller et al., 2009; Miller and Raison, 2016) and is associated with increased cardiovascular and all-cause mortality (Proctor et al., 2015). Meta-analyses of cross-sectional studies have reported that depressed patients have higher mean concentrations of inflammatory markers such as C-reactive protein (CRP) and interleukin 6 (IL-6) in peripheral blood compared with controls (Haapakoski et al., 2015; Howren et al., 2009; Leighton et al., 2018). Longitudinally, higher levels of inflammatory markers in childhood are associated with risks of depression and persistent depressive symptoms subsequently in early-adulthood (Khandaker et al., 2014, 2018). A recent systematic review and meta-analysis reported that about a quarter of depressed patients have evidence of low-grade inflammation, defined as circulating CRP levels >3 mg/L (Osimo et al., 2018). However, the majority of included studies were case-control by design, and based on hospital-treated or treatment-resistant working-age adults (Raison et al., 2013; Uher et al., 2014; Wium-Andersen et al., 2013; Wysokinski et al., 2015), which increases the possibility of selection bias and confounding by comorbid inflammatory physical illness or lifestyle factors. General population-based studies examining the prevalence of low-grade inflammation in depression are scarce, and to our knowledge, no population-based study has examined the prevalence of inflammation in depression in young adults. Studies conducted on younger populations may be beneficial as participants may be less affected by chronic physical illness or lifestyle factors.

People with depression who have evidence of inflammation may represent a high-risk group for cardiometabolic disorders. Co-morbidity of depression with cardiovascular diseases (CVD) (Anda et al., 1993), type 2 diabetes mellitus (T2DM) (Roy and Lloyd, 2012) and obesity (Luppino et al., 2010) is well established, and there is evidence for genetic overlaps between depression, inflammation and obesity (Milaneschi et al., 2017). Inflammation is associated with insulin resistance (IR) (Belgardt et al., 2010), CVD (Danesh et al., 2008) and T2DM (Rader, 2007). We recently reported that inflammation could be a shared mechanism for CVD and depression using MR analysis of UK Biobank data (Khandaker et al., 2019a, 2019b). Depression is associated with cardiometabolic risk factors, such as obesity, altered lipid profile and IR in working age adults (Martin et al., 2016; Timonen et al., 2006). There is also some evidence for these associations in younger adults, though they may be explained by sociodemographic and lifestyle factors (Perry et al., 2020). It has been proposed that “immuno-metabolic depression” may represent a particular ‘sub-type’ of depression characterised by distinct symptom profile (e.g., increased appetite and weight gain) and increased inflammatory and cardiometabolic markers (Lamers et al., 2018; Penninx et al., 2013; Simmons et al., 2018). However, it remains unclear how far back in the life-course an altered immuno-metabolic signature may be detectable.

Based on data from the Avon Longitudinal Study of Parents and Children (ALSPAC), a general population-representative prospective birth cohort from the UK, we have examined the prevalence of low-grade inflammation (serum hsCRP levels >3 mg/L) in participants meeting the ICD-10 criteria for current depressive episode at age 18 years. We have compared cardiometabolic risk between depression with and without evidence of inflammation using a range of established cardiometabolic risk factors at age 18 years and body mass index (BMI) in childhood at ages 9 and 13 years. We hypothesized that depression with evidence of inflammation would be associated with greater cardiometabolic risk both cross-sectionally and longitudinally.

## 2 Methods

### 2.1 Description of cohort and sample

The Avon Longitudinal Study of Parents And Children (ALSPAC) birth cohort initially recruited 14,541 pregnant women resident in county Avon, a geographically defined region in southwest of England, with expected dates of delivery 1 st April 1991 to 31 st December 1992, resulting in 14,062 live births (Boyd et al., 2013; Fraser et al., 2013). Detailed information about the ALSPAC cohort can be found on the study website (http://www.bristol.ac.uk/alspac). The study website contains details of all available data through a fully searchable “data dictionary” (http://www.bris.ac.uk/alspac/researchers/data-access/data-dictionary/). Parents completed regular postal questionnaires about all aspects of their child’s health and development from birth. Since age 7, the children attended an annual assessment clinic during which they participated in various face-to-face interviews and physical tests.

The current study is based on all participants with a measure of the outcome and exposures, after removing participants with CRP > 10 (Khandaker et al., 2014), to minimize the potential confounding effect of ongoing or recent inflammatory disease/infection, or treatment for such conditions. The total sample included 2918 complete cases at age 18 years (mean age = 17.8; SD = 0.38). See Supplementary Fig. 1 for a flow-chart of participants in the study. The authors assert that all procedures contributing to this work follow the ethical standards of the relevant national and institutional committees on human experimentation and with the Helsinki Declaration of 1975, as revised in 2008. All procedures involving human subjects/patients were approved by

**Fig. 1:**
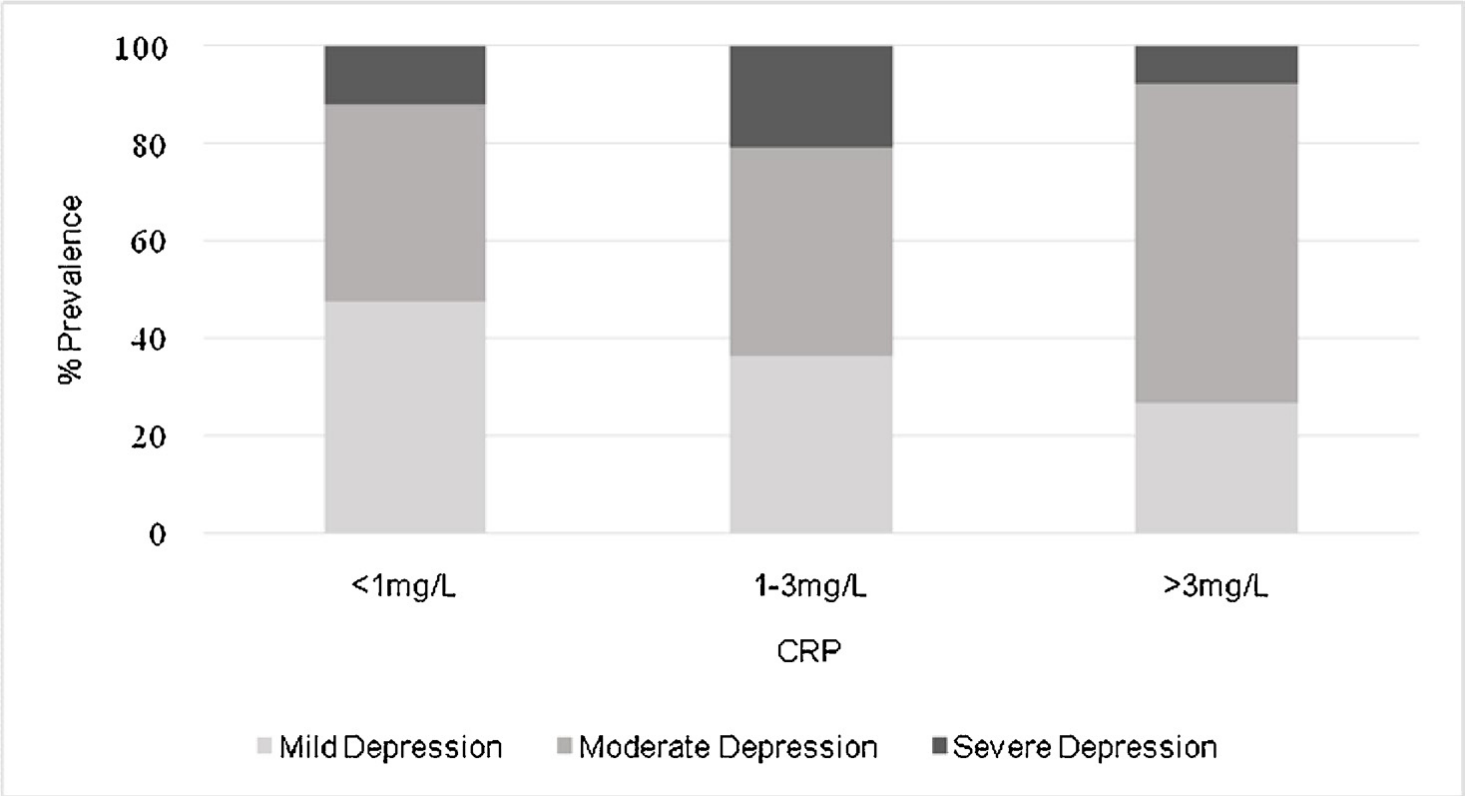
Cases of Mild, Moderate and Severe Depression at Age 18 years Grouped by Serum CRP Level.

the ALSPAC Law and Ethics Committee, and Local Research Ethics Committees. All participants provided informed consent.

### 2.2 Outcome

#### 2.2.1 Measurement of high sensitivity CRP at age 18 years

In total, 3287 participants provided a blood sample. Samples were frozen at −80 °C. The measurements were assayed in 2008 after a median of 7.5 years in storage. There was no evidence of freeze-thaw cycles during storage period. CRP was measured by automated particle-enhanced immunoturbidimetric assay (Roche UK, Welwyn Garden City, UK). All assay coefficients of variation were <5%. The minimum detection limit for CRP was 0.03 mg/L. Twenty-nine participants (0.6 % of the sample) were below this limit and were assigned values of 0.01 (n = 16) and 0.02 (n = 13); they were also included in the analysis. In the total sample, CRP values ranged from 0.01–176.10 mg/L (32 subjects had CRP levels >10 mg/L). Participants with CRP > 10 mg/L (n = 32) were excluded due to the risk of confounding by acute inflammatory state (e.g. infection). We defined evidence of low-grade inflammation as serum CRP>3 mg/L, and no inflammation as CRP<3 mg/L, as is most commonly used in the literature to define low-grade inflammation (Osimo et al., 2018). In addition, we treated CRP as a categorical variable. We created three groups based on CRP levels (<1 mg/L; 1–3 mg/L; >3 mg/L) according to the American Heart Association (AHA) and Centers for Disease Control and Prevention (CDC), USA, guidelines on categorising high-sensitivity CRP levels for cardiovascular studies (Pearson et al., 2003). See Supplementary Methods for further information on measurement of CRP.

#### 2.2.2 Assessment of depression at age 18 years

The computerised version of the Clinical Interview Schedule Revised (CIS-R) was self-administered by cohort participants in assessment clinics at mean age 17.8 years (SD = 0.38). The CIS-R is a widely used, standardized tool for measuring common mental disorders in large community samples (Lewis et al., 1992). The CIS-R is a fully structured assessment, suitable for trained social survey interviewers and does not require any expert knowledge on the part of the interviewers. As such, it can also be administered using personal computers in which the subjects self-complete the questionnaire (Lewis, 1994). The CIS-R elicits responses to depressive symptoms experienced in the preceding week, and provides a diagnosis of current depressive episode according to ICD-10 criteria (WHO, 1992). ICD-10 current depressive episode was used as the main binary outcome measure. In sensitivity analysis, we also used a continuous measure of depressive symptoms score as secondary outcome measure (score range 0–21). This score was created by summing symptom scores for depression, depressive thoughts, fatigue, concentration, and sleep problems also assessed using CIS-R.

### 2.3 Exposures

#### 2.3.1 Cardiometabolic risk factors at age 18 years

##### 2.3.1.1 Glucose-insulin homeostasis

We used the biochemical measurements of fasting plasma glucose (FPG) and fasting insulin (FI). Fasting samples were taken at 0900 after a 10 -h fast (water only). Blood samples were immediately spun, frozen and stored at −80 °C and measurements were assayed within 3–9 months of the samples being taken with no previous freeze-thaw cycles. FPG and FI were measured by an ultrasensitive ELISA (Mercodia, Uppsala, Sweden) automated microparticle enzyme immunoassay. Its sensitivity was 0.07 mU/L, and inter- and intra-assay coefficients of variation were <6%. Insulin resistance was calculated as a continuous measure from FPG and FI by using the computerized, updated version of the Homeostatic Model Assessment (HOMA_2_) for Insulin Resistance (Levy et al., 1998). The algorithm generates a relatively precise measurement of IR taking into account variations in hepatic and peripheral glucose resistance, increases in the insulin secretion curve for plasma glucose concentrations above 10 mmol/L (180 mg/dL) and the contribution of circulating proinsulin (Levy et al., 1998).

##### 2.3.1.2 Lipid Metabolism

We used biochemical measurements of high-density lipoprotein (HDL), low-density lipoprotein (LDL) and triglycerides (TG). Fasting samples were taken at 0900 after a 10 -h fast (water only). Plasma lipid concentrations were measured by modification of the standard Lipid Research Clinics Protocol by using enzymatic reagents for lipid determination. LDL concentration was determined via the Friedwald equation (LDL = total cholesterol – (HDL + TG*0.45)).

##### 2.3.1.3 Smoking and alcohol use

Self-reported data on smoking, alcohol and drug use were collected using a questionnaire. The number of cigarettes/roll ups smoked per day was coded as a binary variable if participant reported smoking ≥1 cigarette per day. Alcohol use was coded as a binary variable if participants reported consuming drinks containing alcohol twice a week or more frequently.

#### 2.3.2 Assessment of BMI at Ages 9, 13 and 18 Years

BMI was calculated as weight (kilograms) divided by height (metre squared) and was measured at various timepoints in a relatively large sample during childhood.

### 2.4 Assessment of potential confounders

We adjusted cross-sectional analyses for sex, ethnicity, paternal social class, physical activity (questionnaire data, binary based on participating in physical exercise e.g. gym attendance, brisk walking or any sports activity at least once per week over the preceding year), smoking (age 18 questionnaire data, binary based upon reporting smoking at least one cigarette per day on average over the preceding year), alcohol use (age 18 questionnaire data, binary, based upon consuming drinks containing alcohol at least twice per week on average over the preceding year), and BMI (age 18 clinic data, continuous). For our longitudinal analysis, we adjusted for sex, ethnicity and paternal social class. We excluded adjustment variables where they were the exposure of interest, since they were already apparent in the statistical model. See Supplementary Data for further information.

### 2.5 Statistical analysis

Exposures that were non-normally distributed were natural log-transformed (all variables except FPG). Resultant variables were standardized (Z-transformed), so the statistical estimations represent the increase in risk of depression (with or without raised CRP) per SD increase in exposure. We completed tests for multi-collinearity of exposures/confounders in a linear regression model. The variance inflation factor for all covariates was between 1.01–1.11, suggesting minimal multi-collinearity. Adjustments were added using the enter method of multiple regression. All statistical analysis was done using IBM SPSS 24.0.

#### 2.5.1 Prevalence of depression with evidence of inflammation

We calculated the proportion of currently depressed participants with “low”, “medium” and “high” inflammation at age 18 years, compared with non-depressed participants. Chi-square tests were used to examine for differences between currently depressed and non-depressed participants in CRP levels, and for associations between severity of depression and CRP levels.

#### 2.5.2 Associations between cardiometabolic risk factors and depression with evidence of inflammation

We used multinomial regression to calculate odds ratios (ORs) and 95 % confidence intervals (C.I.) for the cross-sectional and longitudinal associations between cardiometabolic risk factors and depression with or without evidence of raised CRP. ‘No depression’ was the reference category. We performed likelihood ratio tests to examine whether the estimates for groups were significantly different from each other. Multinomial regression models were adjusted for potential confounders as described above. ORs represent the increase in risk of depression with/without evidence of raised CRP per standard deviation (SD) increase in exposure variable, compared to participants with no depressive episode.

#### 2.5.3 Interactions between CRP and cardiometabolic factors on risk of depression

We performed a sensitivity analysis to examine for any cross-sectional association or interaction between CRP (continuous data) and the cardiometabolic factors described above, on risk of current depression at age 18 years. We performed adjustments as described above. For each cardiometabolic risk factor, we included CRP in the logistic regression model alongside an interaction term (CRP*cardiometabolic risk factor). ORs represent the increase in risk of depression per standard deviation (SD) increase in exposure or interaction variable, compared to participants with no depressive episode.

#### 2.5.4 Interactions between CRP and depression on cardiometabolic risk

We performed a sensitivity analysis to examine for any cross-sectional association of CRP (continuous data) and interaction between CRP and depressive symptom score (continuous data), for the outcomes of cardiometabolic traits (continuous data). Regression models were adjusted as described above. For each continuous cardiometabolic outcome, we included CRP and depressive symptom score in a linear regression model alongside an interaction term (CRP*depressive symptom score). Beta coefficients and 95 % C.I’s represent estimates for the SD increase in continuous cardiometabolic trait per SD increase in exposure or interaction variable.

#### 2.5.5 Multiple testing correction

*p*-values for adjusted regression models for cross-sectional analyses were corrected for multiple testing using the Holm-Bonferroni method (Holm, 1979).

## 3 Results

### 3.1 Sample characteristics and prevalence of depression with evidence of inflammation at age 18 years

Table 1 presents the baseline characteristics of the sample. Out of 2932 participants with data on CRP and depression at age 18 years, 215 met ICD-10 criteria for current depressive episode (7.3 %). The number of participants with mild, moderate, and severe depressive episode was 92 (42 %), 95 (44 %) and 28 (14 %) respectively. Out of 215 cases of current depression, 135 (62.7 %) had CRP <1 mg/L, 57 (26.5 %) had CRP 1−3 mg/L, and 23 (10.7 %) had CRP >3 mg/L (see Table 2; Supplementary Figure 2). There was trend level evidence for differences between depressed and non-depressed participants’ CRP levels (χ^2^ = 5.56; *p* = 0.062), and increased levels of CRP with increasing severity of depression (**χ^2^** = 4.84; *p* = 0.089) (Fig. 1).

**Table 1:** Characteristics of Sample with Data on Depression and CRP at Age 18 Years in the ALSPAC Birth Cohort.

Characteristic	Total Sample	No depression	Depression with Inflammation (CRP >3 mg/L)	Depression without Inflammation (CRP <3 mg/L)
Participants, *n* (% total)	2932 (100)	2717 (93)	23 (1)	192 (6)
Male Sex, *n* (%)	1426 (48)	1368 (50)	3 (13)	55 (29)
Paternal Social Class, *n* (%)				
I & II	1165 (39)	1091 (36)	6 (26)	68 (35)
III – non-manual & manual	1169 (39)	1082 (37)	12 (52)	76 (39)
IV & V	598 (20)	544 (18)	5 (22)	49 (13)
White British Ethnicity, *n* (%)	2873 (98)	2663 (98)	22 (95)	188 (98)
BMI at 18y, mean (SD)	22.66 (3.80)	22.64 (3.76)	25.90 (5.81)	22.69 (4.05)
BMI at 13y, mean (SD)	20.17 (3.16)	20.12 (3.13)	23.08 (3.30)	20.38 (3.18)
BMI at 9y, mean (SD)	17.51 (2.59)	17.48 (2.59)	20.20 (2.26)	17.59 (2.41)
FPG at 18, mean (SD), mmol/L	5.03 (0.47)	5.03 (0.44)	4.95 (0.75)	5.02 (0.83)
HOMA_2_ at 18y, mean (SD)	0.92 (0.73)	0.92 (0.74)	0.93 (0.44)	0.98 (0.72)
CRP at 18y, mean (SD), mg/L	1.09 (1.43)	1.09 (1.43)	4.53 (1.48)	0.82 (0.72)
Smoking at age 18, *n* (%)	211 (7)	185 (7)	2 (9)	24 (13)
Depression score at age 18, mean (SD)	3.09	2.39 (2.91)	12.57 (3.63)	11.98 (3.56)
Physical Activity^a^ at 18y, *n* (%)	1964 (67)	1901 (70)	7 (30)	57 (29)
Alcohol Use^b^ at 18y, *n* (%)	1172 (40)	1059 (39)	11 (48)	102 (53)

^a^Physical activity measured as a binary variable based upon average weekly frequency of at least once per week of going to gym, brisk walking, or any sports activity during the past year.

^b^Alcohol use measured as a binary variable based upon average weekly frequency of at least 2 times per week participant has consumed drinks containing alcohol.

**Table 2:** Prevalence of low, medium and high CRP levels in Participants with and without ICD-10 Current Depressive Episode at age 18 years.

CRP Level (age 18)	No Depressive episode at 18y, *n* (%)	Depressive Episode at 18y, *n* (%)	χ^2^ (df), *p*-value
<1 mg/L	1896 (68.2)	135 (62.7)	5.56 (2), 0.062
1−3 mg/L	728 (26.1)	57 (26.5)	
>3 mg/L	157 (5.6)	23 (10.7)	

### 3.2 Cross-sectional associations between cardiometabolic risk factors and depression with and without evidence of inflammation at age 18 years

Female sex was associated with current depression, both in groups with raised CRP (OR = 7.83; 95 % C.I., 1.78−34.40) and without (OR = 2.35; 95 % C.I., 1.64−3.35), but the association was stronger for cases with raised CRP (Holm-corrected *p-*value for likelihood ratio test <0.001) (Table 3). BMI (OR = 1.13; 95 % C.I., 1.05−1.22), HOMA_2_ (OR = 1.12; 95 % C.I., 1.01−1.26), triglycerides (OR = 2.09; 95 % C.I., 1.35−3.24) and fasting insulin (OR = 1.42; 95 % C.I., 1.01-2.12) were associated with current depression with raised CRP, after adjusting for potential confounders. The likelihood ratio test showed that these effect estimates were significantly different between the two depressed groups (Table 4), though only the *p*-value for the likelihood ratio test for triglycerides survived multiple testing correction.

**Table 3:** Sociodemographic Factors Associated with Depression With and Without Evidence of Inflammation at Age 18 years.

Variable	Unadjusted analysis					Adjusted analysis^a^				
	Depression with Inflammation (CRP >3 mg/L)	Depression without Inflammation (CRP <3 mg/L)	Likelihood Ratio Test			Depression with Inflammation (CRP >3 mg/L)	Depression without Inflammation (CRP <3 mg/L)	Likelihood Ratio Test		
	OR (95 % CI)	OR (95 % CI)	X^2^	p-value	Corrected p-value^d^	OR (95 % CI)	OR (95 % CI)	X^2^	p-value	Corrected p-value^d^
**Female Sex**	6.77 (2.01−22.82)	2.53 (1.83−3.49)	48.26	<0.001	<0.001	7.83 (1.78−34.40)	2.35 (1.64−3.35)	34.56	<0.001	<0.001
**Non-White Ethnicity^b^**	2.81 (0.37−21.49)	0.56 (0.14−2.31)	1.52	0.468	0.468	4.44 (0.56−35.25)	0.86 (0.20−3.62)	1.43	0.490	0.490
**Paternal Social Class^c^**	1.08 (0.88−1.33)	1.07 (0.99−1.15)	3.27	0.195	0.390	1.09 (0.89−1.35)	1.06 (0.99−1.15)	3.31	0.191	0.382

^a^ Regression models were adjusted for sex, ethnicity, paternal social class, smoking, alcohol use, physical activity, BMI (age 18 years).

^b^ The group with British White ethnicity was used as reference; non-white ethnic groups included Bangladeshi, Indian, Pakistani, Chinese, Black Caribbean, Black African, and Other.

^c^ Paternal social class defined as the social class of the father at birth. Class I (professional) was used as reference.

^d^p-values corrected for multiple testing using the Holm-Bonferroni method.

**Table 4:** Cross-sectional Associations of Cardiometabolic Risk Factors with Depression With and Without Evidence of Inflammation at Age 18 Years.

Variable	Unadjusted analysis					Adjusted analysis^a^				
	Depression with Inflammation (CRP >3 mg/L)	Depression without Inflammation (CRP <3 mg/L)	Likelihood Ratio Test			Depression with Inflammation (CRP >3 mg/L)	Depression without Inflammation (CRP <3 mg/L)	Likelihood Ratio Test		
	OR (95 % CI)	OR (95 % CI)	X^2^	p-value	Corrected p-value^b^	OR (95 % CI)	OR (95 % CI)	X^2^	p-value	Corrected p-value^b^
**BMI**	1.14 (1.06−1.21)	1.00 (0.96−1.04)	10.71	0.005	0.040	1.13 (1.05−1.22)	1.00 (0.96−1.04)	8.94	0.011	0.088
**HOMA_2_**	1.17 (1.01−1.36)	1.12 (0.96−1.29)	6.44	0.039	0.234	1.12 (1.01−1.26)	1.01 (0.91−1.18)	5.99	0.046	0.230
**FPG**	0.76 (0.49−1.18)	0.92 (0.78−1.18)	2.38	0.304	1.000	0.92 (0.52−1.66)	1.07 (0.90−1.28)	0.68	0.712	1.000
**FI**	1.46 (1.02−2.10)	1.13 (0.96−1.31)	6.25	0.044	0.234	1.42 (1.01−2.12)	1.15 (0.94−1.33)	2.39	0.030	0.180
**HDL**	0.82 (0.56−1.19)	1.08 (0.93−1.25)	2.12	0.347	1.000	0.75 (0.53−1.08)	0.95 (0.80−1.12)	2.23	0.329	1.000
**LDL**	1.35 (0.87−2.08)	0.99 (0.86−1.14)	1.90	0.387	1.000	1.28 (0.76−2.11)	0.95 (0.80−1.12)	1.38	0.501	1.000
**TG**	1.92 (1.33−2.76)	1.02 (0.88−1.18)	11.47	0.003	0.027	2.09 (1.35−3.24)	0.93 (0.79−1.10)	11.33	0.003	0.027
**Smoking**	1.30 (0.30−5.60)	1.96 (1.24−3.07)	7.43	0.024	0.168	0.84 (0.11−6.55)	2.26 (1.37−3.74)	8.99	0.011	0.088
**Alcohol**	0.86 (0.55−1.21)	1.09 (0.77−1.45)	2.03	0.299	1.000	0.80 (0.50−1.18)	1.08 (0.79−1.43)	2.01	0.301	1.000

BMI = Body Mass Index; HOMA_2_=Homeostatic Model Assessment; FPG = Fasting Plasma Glucose; FI = Fasting Insulin; HDL=High-Density Lipoprotein; LDL = Low-Density Lipoprotein; TG = Triglycerides; PE=’Definite’ Psychotic Experiences. BMI, HOMA_2_, FPG, FI, HDL, LDL, TG coded as continuous variables. Smoking, Drug Use and Alcohol coded as categorical variables.

^a^Regression models were adjusted for sex, ethnicity, paternal social class, physical activity, smoking, alcohol use and BMI (age 18 years) ^b^p-values corrected for multiple testing using the Holm-Bonferroni method.

### 3.3 Longitudinal associations between childhood BMI and depression with and without evidence of inflammation at age 18 years

Higher BMI at both ages 9 (OR = 1.27; 95 % C.I. 1.10−1.48) and 13 (OR = 1.23; 95 % C.I. 1.09−1.38) years was associated with depression with raised CRP at age 18 years (See Table 5).

**Table 5:** Longitudinal associations between Childhood BMI and Depression With and Without Evidence of Inflammation at Age 18 Years.

		Unadjusted Analysis				Adjusted Analysis^a^			
		Depression with Inflammation (CRP >3 mg/L)	Depression without Inflammation (CRP <3 mg/L)	Likelihood Ratio Test		Depression with Inflammation (CRP >3 mg/L)	Depression without Inflammation (CRP <3 mg/L)	Likelihood Ratio Test	p-value
Variable	n	OR (95 % C.I.)	OR (95 % C.I.)	χ²	*p-*value	OR (95 % C.I.)	OR (95 % C.I.)	χ²	
BMI (age 9)	2571	1.31 (1.15−1.49)	1.02 (0.96−1.08)	14.14	0.001	1.27 (1.10−1.48)	1.02 (0.96−1.09)	8.63	0.013
BMI (age 13)	2462	1.23 (1.11−1.37)	1.03 (0.98−1.08)	13.76	0.001	1.23 (1.09−1.38)	1.02 (0.96−1.08)	9.85	0.007

^a^Regression models were adjusted for sex, ethnicity, paternal social class. BMI = Body Mass Index.

### 3.4 Interactions between CRP and cardiometabolic factors on risk of depression

We found trend evidence for an interaction between CRP and BMI (adjusted OR for the interaction term = 1.56; 95 % C.I. 0.98–2.02) and between CRP and triglycerides (adjusted OR for the interaction term = 1.17; 95 % C.I. 0.99–1.38) on risk of current depression. See Supplementary Table 1.

### 3.5 Interactions between CRP and depressive symptom score on cardiometabolic risk

We found trend level evidence for an interaction between CRP and depressive symptom score for the outcome of BMI (adjusted β for the interaction term = 0.05; 95 % C.I. 0.00−0.12). CRP was associated with other cardiometabolic outcomes after controlling for potential confounders: HOMA_2_ (adjusted β = 0.11; 95 % C.I., 0.03−0.19), fasting insulin (adjusted β = 0.10; 95 % C.I., 0.02−0.18), HDL (adjusted β=-0.23; 95 % C.I., −0.31 to −0.15) and triglycerides (adjusted β = 0.22; 95 % C.I., 0.14−0.30). However, there was no evidence for interaction between CRP and depressive symptoms scores in relation to these outcomes.

## 4 Discussion

Using a general population-based birth cohort study, we report that a notable proportion of young adults with current depression have evidence of raised CRP. In our sample, approximately one in ten people meeting ICD-10 criteria for current depressive episode at age 18 had CRP >3 mg/L, while about a quarter had CRP 1−3 mg/L. To our knowledge, this is one of the first studies of the prevalence of low-grade inflammation in young adults enrolled in a population-representative birth cohort. The meta-analytic prevalence of inflammation defined as CRP>3 mg/L is about 27 % (Osimo et al., 2018), and in individual studies this prevalence ranges from 19 to 47% (Uher et al., 2014; Wium-Andersen et al., 2013; Wysokinski et al., 2015). The relatively lower prevalence in our sample could be due to participant age and recruitment method. The majority of previous studies included older participants, increasing the likelihood of comorbid inflammatory physical illness or inflammatory-related lifestyle factors such as smoking (Lee et al., 2012), and many of these studies recruited participants from hospital inpatient or outpatient department, possibly missing milder cases of depression.

We report that participants with a current depressive episode and raised CRP were more likely to be female and have increased cardiometabolic risk factors at age 18 years, and increased BMI in childhood assessed at ages 9 and 13. Our findings may indicate that depression with evidence of inflammation may be more common in females. However, in our sample there were only three male participants who had depression and evidence of inflammation. Therefore, the possibility of type I error due to low statistical power because of small sample size cannot be ruled out. In future, studies with larger samples of depressed cases are required.

Cardiometabolic risk factors were associated with cases of current depression with, but not without, evidence of inflammation. These findings are consistent with the idea that inflammation could be a common mechanism for comorbid depression and cardiometabolic disorders. Comorbidity between depression and cardiometabolic disorders is well known (Vancampfort et al., 2016), and is present even in young people (Lin et al., 2014). We found the evidence for an association of current depression with raised CRP and raised triglycerides, which is consistent with our recent MR study reporting that triglycerides, as well as IL-6 and CRP, could be causally linked to depression (Khandaker et al., 2019a, 2019b). In future, studies should examine mechanisms through which alterations in triglycerides contribute to depression risk to elucidate potential therapeutic targets.

Previous research which considered depression as a single entity, including our own work from the ALSPAC cohort, reported that associations between current depression and cardiometabolic risk factors could be explained by sociodemographic and lifestyle factors including sex, ethnicity, social class and physical activity (Perry et al., 2020). In the current study, we report that evidence for association between a current depressive episode with evidence of inflammation and cardiometabolic risk factors remains after adjustments for potential confounders, which aligns with other research (Rethorst et al., 2014). We also found an interaction between CRP and both BMI and triglycerides on risk of depression, suggesting that inflammation and cardiometabolic factors work together to increase the risk of depression. In addition, we also found some evidence for an interaction between CRP and depressive symptom score for the outcome to BMI, suggesting that inflammation and depressive symptoms may work together to increase BMI. These results are consistent with the concept of a distinct phenotype of ‘immuno-metabolic depression’ (Penninx et al., 2013). Immuno-metabolic depression has previously been linked with distinct depressive symptomatology such as increased appetite disturbance, weight gain (Lamers et al., 2018), and depressed patients with these symptoms have been found to have higher BMI and CRP levels (Lamers et al., 2013) alongside other cardiometabolic alterations such as insulin resistance (Simmons et al., 2018).

Cardiometabolic traits are known to be associated with inflammation (Bulló et al., 2003; Calabro and Yeh, 2008; Jung and Choi, 2014). Our sensitivity analyses showed that CRP was associated with several cardiometabolic factors (HOMA_2_, fasting insulin, triglycerides, and HDL) after adjustments for potential confounders including depressive symptoms. HOMA_2_ is a reliable measure of insulin resistance (Geloneze et al., 2009) and the triad of raised fasting insulin, triglycerides and low HDL can reflect an insulin resistant state (Glueck et al., 2009). Insulin resistance is known to be closely related to inflammatory processes (Chen et al., 2015). Our results suggest that BMI may be mechanistically associated with depression plus evidence of inflammation, while glycaemic traits may arise secondary to the inflammation or by other mechanisms.

Our longitudinal analyses revealed that raised BMI measured in childhood was associated with current depression with raised CRP subsequently at age 18. This finding indicates that underlying biological processes predisposing to cardiometabolic alterations and depression may begin early in the life-course. A growing body of research is beginning to implicate that the cardiometabolic comorbidity associated with depression may originate, in part, from pathogenic processes initiated in fetal development (Goldstein et al., 2019). For example, maternal prenatal glucocorticoid excess and immune pathway abnormalities are associated with sex-dependent impacts on offspring brain circuitry, resulting in depression alongside dysregulation of immune responses and an increased risk of cardiometabolic disorders in adulthood (Domes et al., 2010; Monroe and Harkness, 2005; Pacak et al., 1995).

Strengths of the work include use of data from a prospective general population-representative birth cohort, inclusion of a range of cardiometabolic markers, and longitudinal design. The participants in our study were relatively young, thus reducing the chance of confounding by chronic physical comorbidity or the effects of long-term poor diet, limited exercise, smoking, alcohol use and psychological stress. We were able to adjust for several potential confounders including age, sex, ethnicity, social class, physical activity, alcohol and substance use and BMI.

Limitations of this work include, as with all observational research, residual confounding. We were unable to adjust for psychotropic medication since this data were not available. However, whilst certain antidepressants may predispose to cardiometabolic dysfunction, this is unlikely to affect our longitudinal analysis, since psychotropic medication prescription in childhood is relatively rare. Other immuno-modulatory medications or inflammatory physical illness may also have influenced the CRP data we used in the study. We attempted to address the impact of this potential limitation by excluding participants with CRP >10 mg/L as an indicator of possible acute inflammatory process. Future work may seek to make attempts to more accurately account for the risk of confounding by medication. Whilst we were able to measure a definition of depression meeting ICD-10 criteria, the CIS-R elicits symptoms of depression suffered in the previous week. Therefore, our findings are reflective of a depressed state and it is unclear how our results may be extrapolated to trait depression. Future research may seek to replicate our work with cases of persistent or recurrent depression and evidence of inflammation. Another issue is attrition. Only a subset of the cohort undertook assessment for depression at age 18, and not all those participants had data for the exposures and covariates. Selective attrition of depressed individuals could introduce a bias towards the null, thus our analysis might underestimate the true association between depression and cardiometabolic risk factors. Whilst we included a relatively large overall sample, the numbers of participants with evidence of raised CRP and depression was relatively small. Future research conducted on larger samples of people with depression may be warranted to confirm our findings. Finally, whilst we considered CRP as an indicator of inflammation, because it is the most commonly measured inflammatory biomarker in hospital laboratories, future research may seek to examine a greater range of inflammatory biomarkers.

## 5 Conclusions

In summary, our findings suggest that depression with evidence of inflammation represents a high-risk state for cardiometabolic disorders. Therefore, clinicians should need to proactively assess and manage cardiovascular risk in people presenting with depression who show evidence of inflammation to improve long-term health outcomes. Evidence for the longitudinal association with childhood BMI indicates that cardiometabolic alterations in young adults presenting with depression are unlikely to be fully explained by reverse causality or by confounding from lifestyle factors. The findings are consistent with the idea that inflammation could be a shared mechanism for depression and cardiovascular disease.

## Funding statement

BIP is funded by National Institute for Health Research (NIHR), (Doctoral Research Fellowship, DRF-2018-11-ST2-018) for this research project. This paper presents independent research funded by the National Institute for Health Research (NIHR). The views expressed are those of the author(s) and not necessarily those of the NHS, the NIHR or the Department of Health and Social Care. GMK acknowledges funding support from the MQ: Transforming Mental Health (Data Science Award; grant code: MQDS17/40), the Wellcome Trust (Intermediate Clinical Fellowship; grant code: 201486/Z/16/Z), the Medical Research Council (MICA: Mental Health Data Pathfinder; grant code: MC_PC_17213), and the BMA Foundation J Moulton Grant (2019). The UK Medical Research Council and Wellcome (Grant ref: 102215/2/13/2) and the University of Bristol provide core support for ALSPAC. This publication is the work of the authors and the authors will serve as guarantors for the contents of this paper. A comprehensive list of grants funding is available on the ALSPAC website: http://www.bristol.ac.uk/alspac/external/documents/grantacknowledgements.pdf/. This research was specifically funded by The Wellcome Trust (Grant no. 08426812/Z/07/Z) (Awardee: Dr Golam M Khandaker).

## Author contribution

Based on existing ALSPAC cohort data, this specific study design and analysis were conceived by GMK. Analysis was conducted by BO and BIP. The manuscript was prepared by BIP. GMK and PBJ provided oversight and revised the manuscript.

## Data availability

Data was obtained following a successful application to the ALSPAC executive committee.

## Declaration of Competing Interest

None.
