# Supplementary material for: Cardiometabolic risk in young adults with depression and evidence of inflammation: A birth cohort study
Source: Psychoneuroendocrinology. 2020 Jun;116:104682. doi: 10.1016/j.psyneuen.2020.104682 (PMC7301151; doi:10.1016/j.psyneuen.2020.104682)
Supplement: Supplementary file 1 [file mmc1.docx]

**Depression with Inflammation in Young Adults is Characterised by Increased Cardiometabolic Risk: A Birth Cohort Study**

*Perry, B.I. et al*

**Supplementary Data**

**Supplementary Methods**

**Supplementary Figure 1: Flow-Diagram of Included Participants**

Glucose/Insulin Homeostasis

*n* = 3,232

## *n* with data on exposures and outcome at age 18y

## *n* cases of outcome

## *n* with data on exposures at age 18y

Complete Cases

*n* = 2918

Cases with CRP>10 removed from analysis

*n* = 32

Depression with Inflammation
 *n* = 23

Smoking & Alcohol Use

*n =* 3.969

Lipid Homeostasis

*n* = 3,286

CRP

*n* = 3,287

Undertook CIS-R

*n* = 4,561

Depression without Inflammation

*n* = 192

**Supplementary Results**

**Supplementary Figure 2: Prevalence of Low, Medium and High CRP Levels in Cases of Depressive Episode at Age 18 years**

<1mg/L

1-3mg/L

>3mg/L

<1mg/L

1-3mg/L

>3mg/L

<1mg/L

1-3mg/L

>3mg/L

>3mg/L

1-3mg/L

<1mg/L

**Supplementary Table 1: Interactions between Cardiometabolic Risk Factors and CRP on Cross-Sectional Depression Risk at Age 18 Years**

| **Exposure (age 18y)** | **Adjusted Odds Ratio (95% C.I)^1^** | **P-Value** |
| --- | --- | --- |
| BMI | 1.01 (0.97-1.05) | 0.659 |
| CRP | 0.83 (0.67-1.02) | 0.079 |
| BMI*CRP | 1.56 (0.98-2.02) | 0.063 |
| HOMA_2_ | 1.09 (0.93-1.28) | 0.275 |
| CRP | 0.93 (0.79-1.12) | 0.478 |
| HOMA_2_*CRP | 1.11 (0.94-1.31) | 0.209 |
| FPG | 1.05 (0.89-1.24) | 0.557 |
| CRP | 0.96 (0.82-1.15) | 0.751 |
| FPG*CRP | 1.14 (0.90-1.34) | 0.294 |
| FI | 1.12 (0.96-1.31) | 0.163 |
| CRP | 0.93 (0.78-1.12) | 0.442 |
| FI*CRP | 1.14 (0.97-1.33) | 0.106 |
| HDL | 0.93 (0.80-1.08) | 0.312 |
| CRP | 0.95 (0.80-1.13) | 0.569 |
| HDL*CRP | 1.07 (0.93-1.24) | 0.338 |
| LDL | 1.02 (0.88-1.19) | 0.764 |
| CRP | 0.97 (0.82-1.14) | 0.692 |
| LDL*CRP | 0.99 (0.84-1.16) | 0.855 |
| TG | 1.07 (0.91-1.25) | 0.415 |
| CRP | 0.92 (0.77-1.10) | 0.375 |
| TG*CRP | 1.17 (0.99-1.38) | 0.061 |
| Smoking | 2.16 (1.36-3.45) | 0.001 |
| CRP | 0.99 (0.83-1.19) | 0.959 |
| Smoking*CRP | 0.68 (0.40-1.16) | 0.155 |
| Alcohol | 1.08 (0.88-1.21) | 0.516 |
| CRP | 0.99 (0.78-1.12) | 0.887 |
| Alcohol*CRP | 0.81 (0.60-1.43) | 0.435 |

^1^Adjusted for sex, ethnicity, paternal social class, physical activity, smoking, alcohol use

**Supplementary Table 2: Interactions between CRP and Depression on Cross-Sectional Cardiometabolic Risk at Age 18 Years**

| **Outcome (age 18y) /**  **Exposure (age 18y)** | **Adjusted β (95% C.I)^1^** | **P-Value** |
| --- | --- | --- |
| **BMI** | | |
| Depressive Symptom Score | 0.05 (0.00-0.11) | 0.069 |
| CRP | 0.03 (-0.05-0.07) | 0.443 |
| Interaction | 0.05 (0.00-0.12) | 0.078 |
| **HOMA_2_** | | |
| Depressive Symptom Score | 0.00 (-0.01-0.02) | 0.733 |
| CRP | 0.11 (0.03-0.19) | 0.009 |
| Interaction | 0.01 (-0.01-0.03) | 0.117 |
| **Fasting Plasma Glucose** | | |
| Depressive Symptom Score | 0.01 (-0.02-0.02) | 0.239 |
| CRP | -0.03 (-0.12-0.03) | 0.264 |
| Interaction | 0.01 (-0.01-0.02) | 0.303 |
| **Fasting Insulin** | | |
| Depressive Symptom Score | 0.01 (-0.01-0.02) | 0.443 |
| CRP | 0.10 (0.02-0.18) | 0.015 |
| Interaction | 0.02 (-0.03-0.05) | 0.401 |
| **HDL** | | |
| Depressive Symptom Score | -0.01 (-0.02-0.01) | 0.109 |
| CRP | -0.23 (-0.31- -0.15) | <0.001 |
| Interaction | 0.01 (-0.01-0.02) | 0.347 |
| **LDL** | | |
| Depressive Symptom Score | 0.01 (-0.01-0.02) | 0.229 |
| CRP | -0.03 (-0.12-0.05) | 0.458 |
| Interaction | 0.01 (-0.01-0.03) | 0.098 |
| **Triglycerides** | | |
| Depressive Symptom Score | 0.00 (-0.01-0.01) | 0.961 |
| CRP | 0.22 (0.14-0.30) | <0.001 |
| Interaction | 0.01 (-0.01-0.02) | 0.734 |

^1^Adjusted for sex, ethnicity, paternal social class, physical activity, smoking, alcohol use
